# Ophthalmologists’ Evaluation by Physician Review Websites—Do Only Soft Skills Matter? A Cross-National Analysis of over 70,000 Patient Reviews

**DOI:** 10.3390/healthcare13131548

**Published:** 2025-06-28

**Authors:** Wojciech Janikowski, Agnieszka Jóźwicka, Janusz Skrzypecki, Aleksandra Pyziak-Skupień, Jacek P. Szaflik, Joanna Przybek-Skrzypecka

**Affiliations:** 1 Department of Ophthalmology, Medical University of Warsaw, 03-709 Warsaw, Poland; s081028@student.wum.edu.pl (W.J.); s081031@student.wum.edu.pl (A.J.); jacek.szaflik@wum.edu.pl (J.P.S.); 2 Department of Experimental Physiology and Pathophysiology, Medical University of Warsaw, 02-097 Warsaw, Poland; janusz.skrzypecki@wum.edu.pl; 3 Independent Researcher, 40-752 Katowice, Poland; aleksandra.ewa.pyziak@gmail.com; 4 SPKSO Ophthalmic University Hospital, 03-709 Warsaw, Poland

**Keywords:** ophthalmologist, physician rating websites, soft skills, patient satisfaction, patient rating, online rating

## Abstract

Background: Physician rating websites (PRWs) are increasingly used by patients to assess healthcare providers, yet little is known about the factors influencing patient feedback in ophthalmology across different healthcare systems. Objective: This study aimed to identify variables associated with patient reviews of ophthalmologists, with a particular focus on soft skill-related feedback, and to compare trends between Polish and British healthcare systems. Methods: We analyzed data on 461 verified ophthalmologists—261 from ZnanyLekarz.pl (Poland) and 200 from Doctify.com (UK)—collected between March and July 2024 with the highest number of reviews. Physician characteristics, including sex, academic title, years of experience, practice setting, specialization, and online presence, were examined. Review content was categorized into soft skills, professional abilities, or both. Statistical and multivariate analyses were performed to determine factors influencing (a) soft review frequency and (b) the profile of a top-rated ophthalmologist, defined as one with the highest overall ratings and a minimum of 100 reviews. Results: We analyzed a total of 70,176 patient reviews—55,786 from a Polish physician rating website (PRW) and 14,390 from a British PRW. Polish ophthalmologists received significantly more reviews than their British counterparts, with a median of 141 reviews per physician compared to 44 in the UK (*p* < 0.001). Feedback focused on soft skills accounted for 74% of Polish reviews and 59% of British reviews. Key predictors of soft skill-focused reviews included female gender, higher average ratings, practice in pediatric ophthalmology, and affiliation with the private sector. In contrast, fewer soft reviews were associated with area of surgical expertise, public sector employment, and practicing in the UK. Academic title also influenced the content of reviews: physicians holding PhDs received more feedback focused on soft skills, while full professors received less and years of experience had no significant effect. In the multivariate model assessing predictors of being a top-rated ophthalmologist for the whole cohort, each additional review containing substantive content or soft skill-related feedback increased the odds by 29% and 14%, respectively (*p* < 0.001 for both). Conclusions: Patient reviews are largely influenced by perceived interpersonal qualities rather than professional credentials or clinical experience. The active solicitation of patient feedback, as implemented on Polish physician rating platforms, results in over three times the volume of reviews compared to the British approach, which relies on passive voluntary submission without direct prompts.

## 1. Introduction 

In today’s digital era, the Internet has become the dominant source for health information and a key tool in shaping patient choices. Physician rating websites (PRWs) are increasingly used by patients to evaluate healthcare providers, particularly in terms of service quality, accessibility, and patient experience [1,2,3,4]. These platforms influence how patients select physicians and provide feedback based on factors such as review sentiment, physician gender, experience, communication, diagnostic accuracy, and treatment outcomes [4,5,6,7,8,9].

While PRWs have been studied in various countries [10,11,12,13] and specialties [5,6,7,8,9], including refractive surgery [14], there is limited research on their use in general ophthalmology. Additionally, little is known about how PRWs function in different healthcare systems, particularly in Poland, where research is sparse. Cross-national comparisons remain underexplored, especially in terms of how platform design and healthcare context affect review behavior. Notably, healthcare systems differ significantly between countries, potentially shaping how patients interact with PRWs. The Polish system is characterized by a mix of public and private care, with long wait times in the public sector often prompting patients to seek private services. In contrast, the UK’s National Health Service (NHS) offers universal public healthcare, with private options also available, but these are used less frequently so far. These systemic differences may influence not only patient expectations and satisfaction but also motivations to leave reviews—whether to express gratitude, frustration, or guide others in navigating access to care. Furthermore, the most well-known Polish PRWs actively encourage patients to leave feedback—typically via email or text message within 24 h of their appointment—whereas their British counterparts leave it entirely up to the patients to decide whether or not to comment on their visit.

This study aims to identify key factors shaping patient opinions of ophthalmologists by comparing two PRWs, namely Znany Lekarz (Poland) and Doctify (UK). We analyzed how physician attributes (e.g., range of services, experience, communication skills) and platform-driven review solicitation strategies influence both the number and content of reviews. The study also investigates how national healthcare structures may shape patient engagement with PRWs and the feedback they provide.

## 2. Methods 

This study analyzed data from 261 ophthalmologists on ZnanyLekarz.pl and 200 on Doctify.com, collected between March and July 2024. Only verified medical doctors’ profiles were included (excluding optometrists, opticians, and orthoptists mistakenly reviewed by patients as ophthalmologists). To ensure we assessed only highly rated doctors, we set a minimum threshold of 50 reviews on ZnanyLekarz and 15 on Doctify, reflecting the lower volume of reviews on the latter British-based platform. “Top-rated ophthalmologists” were defined as those with the highest overall ratings and at least 100 patient reviews on the aforementioned platforms.

Key variables collected included physician sex, years of experience (sourced from ZnanyLekarz, Nil.org.pl, and Doctify), academic title (MD, PhD, or Professor), presence and number of research publications (from PubMed, Google Scholar, personal websites), and employment in the private or public healthcare sector or both. Practice location data included the country, city, and population size, categorized as capital cities, cities with over 100,000 residents, or smaller municipalities.

Additional variables included the scope of services (e.g., treatment of pediatric patients, surgical involvement in ocular or plastic/esthetic eye surgery) and social media presence (Instagram, Facebook, personal websites).

Each physician’s average star rating and total number of reviews were recorded retrospectively. Review content was categorized into three groups, including (1) soft skills, (2) professional abilities, and (3) a combination of both further called “mixed review”. We developed a simplified coding scheme inspired by the domains of the PSQ-18 and SERVQUAL questionnaires. From the PSQ-18, we focused on "soft skills" such as “interpersonal manner”, “communication”, and “time spent with the doctor”. From SERVQUAL, we incorporated elements of “assurance”, “empathy”, and “responsiveness”. We defined “professional abilities” as aspects related to correct diagnosis and treatment, aligning with the reliability dimension of SERVQUAL (“the ability to perform the promised service dependably and accurately”). To identify soft skills, we looked for key terms and expressions such as friendly, sympathetic, trustworthy, kind, nice, approachable, warm, empathic, eager to help, helpful, friendly atmosphere, “have time for me”, calm, no rush, compassionate, thoughtful, pleasant personality, cordial, polite, genial, or semantic synonyms. For professional competencies (substantive reviews), we searched for keywords including proper treatment, outcome, favourable outcome, diagnosis, surgical outcome, and symptoms improvement.

Two independent raters (WJ, AJ) coded all patient comments. In most cases, their classifications were consistent. When discrepancies arose or the proper classification was uncertain, a third reviewer (JP-S) adjudicated the final classification.

### Statistical Analysis 

Numeric variables were presented with the mean ± standard deviation or median and interquartile range (IQR) depending on the normality of distributions. Nominal variables were presented with the absolute and relative number of observations. The Shapiro–Wilk test, skewness, and kurtosis were used to verify distribution normality. Levene’s test was employed to verify variance homogeneity. Comparisons of numeric variables between groups were performed with the t-Student test, t-Walch test, or Mann–Whitney U test, as appropriate. Nominal variables were compared between groups with Pearson’s chi-squared test or Fisher’s exact test, as appropriate. Two-step linear regression analysis was employed to identify factors associated with the number of soft reviews. A cut-off at the level of *p* = 0.157 [15] was used for the pre-selection of variables into the multivariate step. Furthermore, a stepwise selection algorithm was run for a final selection. Multivariate model fit was verified with R2 and adjusted R2. Two-step logistic regression analysis was also employed to identify factors associated with the total number of reviews > 100. Cut-off at the level of *p* = 0.250 [16] was used for the pre-selection of variables into the multivariate step. Furthermore, the stepwise selection algorithm was run for final selection. Multivariate model fit was verified with Nagelkerke R2. All calculations assumed significance if *p* < 0.05. The analysis was run in R software (version 4.4.2, https://community.chocolatey.org/packages/r.project (accessed on 23 June 2025), Chocolatey Software, Inc., Seattle, WA, USA).

## 3. Results

### 3.1. Demographics

Data from 461 ophthalmologists were analyzed, with demographic characteristics summarized in Table 1. Women represented 46.4% of the overall cohort; however, gender distribution varied significantly between countries. In Poland, women constituted the majority (66.7%), whereas in the UK, they were a minority (20.0%) (*p* < 0.001). In both countries, the most common practice locations were cities with populations over 100,000—80.5% in Poland and 39.5% in the UK—although the distribution in the UK was more evenly spread across different city sizes (*p* < 0.001).

UK ophthalmologists had significantly more years of professional experience compared to their Polish counterparts (25.25 vs. 20.70 years; *p* < 0.001). The distribution of academic titles also differed between the two countries. While the MD degree was the most common in both cohorts (Poland: 69.0%; UK: 83.0%; *p* < 0.001), Poland had a higher proportion of physicians with PhDs (30.7% vs. 12.5%), whereas full professors were more frequently evaluated in the UK (4.5% vs. 0.4%).

Scientific activity was significantly higher among UK ophthalmologists, with 92.2% having at least one PubMed-indexed publication compared to 41.4% in Poland (*p* < 0.001). The median number of publications was also higher in the UK (7 vs. 0). Private practice was nearly universal across both countries (99.8% overall); however, Polish ophthalmologists were less likely to work simultaneously in the public sector compared to their British counterparts (50.4% vs. 76.0%; *p* < 0.001).

### 3.2. Additional Professional Activities

Additional professional activities are presented in Table 2. Surgical procedures were significantly more common among UK ophthalmologists compared to their Polish counterparts (97.0% vs. 45.7%; *p* < 0.001). In contrast, pediatric ophthalmology was far more prevalent in Poland (89.3%) than in the UK (12.6%; *p* < 0.001). Esthetic medicine procedures were relatively uncommon in both groups, with a similar representation (Poland: 13.5%; UK: 10.0%; *p* < 0.001).

### 3.3. Reviews—Overall Structure

The overall distribution of ratings is presented in Table 3. A total of 70,176 patient reviews were analyzed, including 55,786 from ZnanyLekarz and 14,390 from Doctify. Polish ophthalmologists had a significantly higher median number of reviews than their British counterparts (141 vs. 44; *p* < 0.001) and slightly higher average star ratings (5.00 vs. 4.97). Soft skill-related feedback was over three times more frequent in the Polish group, comprising 74% of all reviews, compared to 59% in the UK (*p* < 0.001). Additionally, reviews concerning pediatric appointments were significantly more common in Poland (median 14 vs. 0; *p* < 0.001).

### 3.4. Determinants of Soft Review Volume

As shown in Table 3, all physicians received at least one soft skill-related review. Table 4 summarizes the factors associated with the number of such reviews. Female ophthalmologists received more soft skill reviews than their male counterparts, and those practicing in cities with over 100,000 inhabitants received more than those based in capital cities. A higher number of soft skill reviews was also associated with providing pediatric consultations and having a higher average star rating.

**Figure 1:**
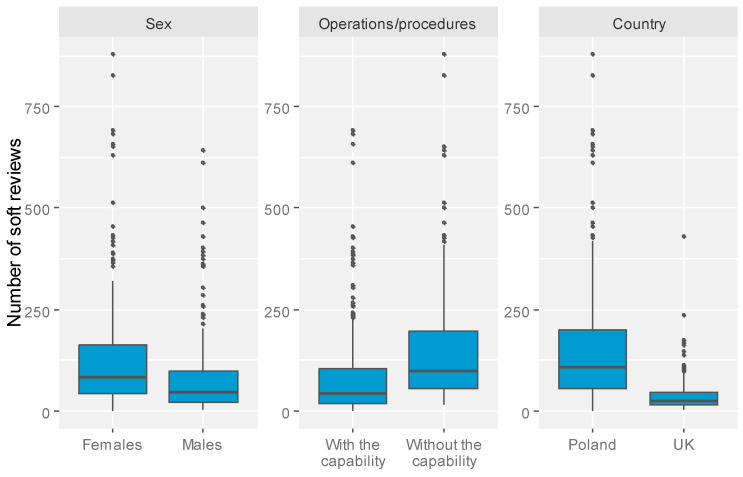
Boxplot charts presenting the distribution of the number of soft skill reviews by significant factors (based on multivariate linear regression model).

In contrast, employment in the public sector, a strong scientific background—as indicated by the number of PubMed-indexed publications—and frequent performance of surgical procedures were all linked to a lower proportion of soft skill mentions in patient feedback. The most pronounced decrease was observed among UK-based ophthalmologists, who received significantly fewer soft skill reviews compared to their Polish peers. Interestingly, the total number of reviews had minimal influence on the number of soft skill comments (Figure 1).

Multivariate analysis confirmed that female gender and higher average star ratings were independent predictors of receiving more soft skill reviews, whereas surgical specialization and practicing in the UK were independently associated with receiving fewer (Table 4).

### 3.5. Top-Rated Ophthalmologists

In the next step, we analyzed physicians with the highest number of favorable reviews and the factors associated with this outcome. Table 5 presents the characteristics of ophthalmologists who received more than 100 positive reviews compared to those with 100 or fewer. Factors strongly associated with a higher number of favorable reviews included female gender, practicing in cities with over 100,000 inhabitants, providing pediatric consultations, and working within the Polish healthcare system. In contrast, physicians with fewer reviews were more often those practicing in capital cities or in smaller municipalities (under 100,000 residents), employed in the public sector, performing surgical procedures, and working within the UK healthcare system.

In the regression analysis, pediatric sub-specialization (OR = 6.44; *p* < 0.001), female gender (OR = 1.54; *p* = 0.023), and practicing in a large city (OR = 1.92; *p* = 0.006) were significantly associated with a higher number of patient reviews. Additionally, a greater number of both substantive and soft skill-related reviews were strong predictors of overall review volume. Conversely, employment in the public sector (OR = 0.48; *p* < 0.001), the frequent performance of procedures (OR = 0.49; *p* < 0.001), and practicing in the UK versus Poland (OR = 0.17; *p* < 0.001) were associated with fewer reviews. Notably, in the multivariate model, being an ophthalmic surgeon was independently associated with an increased likelihood of receiving a high number of reviews. Academic titles, years of professional experience, and involvement in esthetic procedures did not show any significant association with review volume (Table 6).

## 4. Discussion

This study analyzed a large cohort of ophthalmologists (*n* = 461) and a substantial number of patient reviews (*n* = 70,176) to identify factors influencing both the nature and the volume of feedback, with particular emphasis on soft skill-related reviews, which constituted the majority. The most impactful factor was the healthcare system in which the physician practices. Polish ophthalmologists received more reviews overall and more feedback emphasizing interpersonal qualities. This may reflect differences in platform design: the Polish website actively encourages patients to leave reviews via automated reminders, whereas the British one relies on passive methods such as physician prompts or voluntary engagement. “Znany Lekarz” sends both a text message (SMS) and an email 24 h after the visit, prompting patients to leave a review—unlike Doctify, which leaves the decision entirely to the patient without any reminders. Additionally, the message from “Znany Lekarz” begins with the doctor’s name and includes a polite yet somewhat suggestive request to leave a review, which may feel slightly imposing. Prior research confirms that proactive review solicitation increases both the frequency and positivity of reviews [17,18]. Regression analysis showed that pediatric sub-specialization, female gender, and working in large cities were associated with a higher number of reviews. Conversely, physicians working in the public sector, those performing surgical procedures, and those practicing in the UK received fewer reviews. These findings align with broader observations on how context and sub-specialization shape patients’ feedback. To the best of our knowledge, this is the first large-scale, cross-sectional study to encompass all subspecialties of ophthalmology from the patient perspective. While isolated reports on refractive surgeons and oculoplastic units exist [14,19], none have taken such a comprehensive approach.

At the outset, it is important to highlight the disparity between the British and Polish ophthalmology systems. In the UK, surgical expertise is an integral component of the residency curriculum for all ophthalmology trainees. In contrast, Poland lacks a formal regulatory framework governing the initiation of surgical practice. This creates a “grey zone” regarding when and how ophthalmologists transition into surgical roles and gain recognition as surgical experts. In the UK, primary eye care is provided mainly by certified optometrists, opticians, and orthoptists. As a result, at least in theory, only medical, surgical, or more complex cases are handled by ophthalmologists. Although optometrists in Poland have recently gained certain professional entitlements, the majority of primary ocular care is still provided by ophthalmologists. Additionally, the British system offers structured fellowship programs that provide comprehensive subspecialty training—for example, in pediatric ophthalmology. In Poland, however, the majority of ophthalmologists enter the private sector following residency, where they typically practice as general ophthalmologists treating both adult and pediatric patients. The abovementioned factors might partly explain the stark contrast in the proportion of medical doctors offering pediatric ophthalmology consultations—with 90% in Poland versus just 13% in the UK. Given the total number of registered ophthalmology specialists—3447 in the UK and 4977 in Poland—we proportionally analyzed a larger share of Polish ophthalmologists compared to their British counterparts. Finally, it is worth noting that the British system places significantly greater emphasis on scientific research, particularly as a criterion for admission into ophthalmology residency programs.

Since soft skill reviews made up the majority of all reviews— with a median of 107 out of 144 in the Polish group and 26 out of 44 in the British group—we chose to focus primarily on the factors influencing this type of feedback. Among all variables, the physician’s average rating was the strongest predictor of soft reviews. Positive experiences often lead to high ratings, and the existing literature shows that traits such as empathy, attentiveness, and clear communication are strongly linked to favorable feedback [20,21,22,23,24,25,26]. These qualities help build trust, a central component of the doctor–patient relationship. Gender played another key role in shaping review content. Female ophthalmologists received more reviews, particularly soft ones, possibly reflecting different communication styles or patient expectations. Studies suggest that women physicians tend to prioritize emotional support and patient-centered dialog [27,28,29], which may be more frequently recognized by patients in their online reviews.

Surgical expertise was associated with a lower number of both soft skill-focused reviews and overall patient opinions. This may reflect a tendency among surgical patients to prioritize clinical outcomes over interpersonal aspects of care. However, prior studies emphasize that interpersonal skills remain important even in surgical contexts [19,30]. In contrast, esthetic medicine procedures—particularly involving the eye and orbit—were linked to more soft reviews, highlighting the importance of bedside manner and clarity in these fields [31,32,33]. This trend may also be linked to the fully private sector, where esthetic medicine is commonly practiced. We observed that the workplace setting significantly influenced review patterns. Ophthalmologists working in public healthcare received fewer soft skill-related reviews compared to those in private practice. This could be attributed to the high urgency or complexity of cases typically seen in the public sector, where clinical outcomes may take precedence over interpersonal aspects of care [34,35,36].

Scientific credentials appeared to be undervalued by patients. Neither holding a professor degree nor the number of PubMed-indexed publications was associated with more favorable reviews. This supports previous findings showing no correlation between academic impact and patient satisfaction [37]. In fact, physicians with a stronger research focus tended to receive fewer comments, particularly on soft skills, possibly reflecting a communication gap or differing patient expectations.

Pediatric ophthalmology sub-specialization emerged as one of the strongest predictors of the total number of reviews as well as soft reviews. Parents actively use PRWs when choosing care for their children [38] and consistently emphasize qualities such as friendliness, patience, and clarity of recommendations [5,14,39]. A large body of the literature confirms the central role of the aforementioned traits in pediatric subspecialties. Strikingly, a large body of evidence in a variety of pediatric subspecialties also suggests this plays a primary role in parents’ perspective [5,9,14,39]. Recognizing these expectations can support pediatric ophthalmologists in fostering stronger patient engagement and optimizing their visibility on digital platforms.

Next, since our primary objective was to assess top-rated ophthalmologists in order to identify key factors driving a high volume of patient feedback, our analysis may be subject to selection bias. By excluding ophthalmologists with fewer than 50 reviews in the Polish system and 15 reviews in the British system, we may have overlooked valuable insights from providers with a smaller number of ratings, thus potentially limiting the generalizability of our findings. It has previously been demonstrated that the average star rating tends to correlate with the number of reviews. Physicians with fewer ratings may receive more polarized feedback, consistent with the existing literature indicating that online reviews are often left by individuals who are either highly satisfied or highly dissatisfied with their experience.

Notably, insight into reviewer profiles would be valuable. However, reviews are submitted anonymously, limiting the analysis of reliable data regarding reviewer demographics such as age, gender, or healthcare literacy. Additionally, we cannot verify whether some reviews are duplicated (e.g., written multiple times by the same individual), fabricated, or authored by the healthcare provider themselves—or by third parties acting on their behalf to boost online visibility. Moreover, with the increasing use of artificial intelligence in multiple sectors, there is growing concern that some reviews may be generated by non-human agents, further complicating verification [40]. However, we know from previous research that online feedback is more frequently submitted by younger individuals who are accustomed to using the internet and sharing opinions since early adulthood. In contrast, older patients—who may have more complex medical histories and theoretically value more a proper diagnosis or effects of treatment—tend to leave reviews less frequently, which could further skew the representation of patient perspectives [41]. Furthermore, the strong involvement of the private sector in Poland may also contribute to polarization in review statistics. As a result, it may not be appropriate to extrapolate these findings to countries with a different balance between public and private healthcare sectors—the UK, for example. However, we intentionally included two countries with differing ocular healthcare structures, as well as the PRW model, to unify our findings and offer a broader perspective.

This study has several limitations. The data collected did not include every ophthalmologist practicing in the UK and Poland. Due to the strenuous task of analyzing over 70,000 reviews, we established a cut-off point for the number of reviews required for a doctor to be included in the analysis. Furthermore, we acknowledge that some feedback may have been lost due to the exclusion of isolated highly polarized reviews. However, this step was necessary to minimize the risk of skewed average star ratings resulting from insufficient review volume. Next, we limited our analysis to a single physician rating platform per country. This decision was based on the selection of platforms that, in our judgment, provided the most robust and multifactorial data for analysis. ZnanyLekarz and Doctify were chosen because no other platforms in their respective countries match the scale, longevity, and recognition. Both are widely known among patients and host an extensive database of physician reviews. Additionally, this cross-national study only includes two countries. Given the structural differences in ophthalmology practices across Europe and the complexity of such an analysis, we believed it would be more coherent and manageable to focus on just two regions.

## 5. Conclusions

In conclusion, this study, encompassing a large cohort of ophthalmologists and patient reviews across two healthcare systems, identified key factors influencing the quantity and nature of online feedback. Pediatric subspecialty, female gender, and urban practice were associated with higher review counts, while public sector, surgical expertise, and UK practice were linked to fewer reviews. Soft skills such as empathy and communication were the strongest predictors of favorable feedback, whereas academic credentials had little influence. Surgical patients prioritize outcomes over bedside manners. Understanding these patterns can help medical professionals enhance their online image on physician rating websites and align their practice with the evolving expectations of patients in the digital age.

## Figures and Tables

**Table 1:** Overview of physician characteristics and country-level differences.

Variable	Total Group	Poland	UK	MD/RR (95% CI)	*p *
Number of physicians, n	461	261	200	−	−
Sex, female, *n* (%)	214 (46.4)	174 (66.7)	40 (20.0)	3.33 (2.49; 4.46)	**<0.001**
City size, *n* (%)					
Capital city	101 (21.9)	33 (12.6)	68 (34.0)	−	**<0.001**
Above 100k citizens	289 (62.7)	210 (80.5)	79 (39.5)		
Other	71 (15.4)	18 (6.9)	53 (26.5)		
Professional experience, years, mean ± SD	22.70 ± 9.74	20.70 ± 10.41	25.25 ± 8.17	4.55 (−6.26; −2.83)	**<0.001 ^1^**
Scientific title, *n* (%)					
MD	346 (75.1)	180 (69.0)	166 (83.0)	−	**<0.001**
PhD	105 (22.8)	80 (30.7)	25 (12.5)		
Professor (full)	10 (2.2)	1 (0.4)	9 (4.5)		
PubMed publications, Any, *n* (%)	285 (62.9)	108 (41.4)	177 (92.2)	0.45 (0.39; 0.52)	**<0.001**
Number of PubMed publications, median (IQR)	1.00 (0.00; 7.00)	0.00 (0.00; 2.00)	7.00 (3.00; 14.50)	−7.00 (−7.00; −5.00)	**<0.001 ^2^**
Public workplace, *n* (%)	283 (61.5)	131 (50.4)	152 (76.0)	0.66 (0.57; 0.77)	**<0.001**
Private workplace, *n* (%)	460 (99.8)	260 (99.6)	200 (100.0)	1.00 (0.99; 1.00)	>0.999

SD—standard deviation; IQR—interquartile range; MD—mean or median difference (Poland vs. UK); RR—relative risk (Poland vs. UK); CI—confidence interval. Numeric variables compared with the t-Welch ^1^ test or Mann–Whitney U test ^2^. Nominal variables compared with Pearson’s chi-squared test. Bold values indicate statistically significant differences (*p* < 0.05).

**Table 2:** Additional clinical and procedural competencies: comparison between Poland and the UK.

Variable	Total Group	Poland	UK	RR (95% CI)	*p *
Surgical procedures, *n* (%)	313 (67.9)	119 (45.6)	194 (97.0)	0.47 (0.41; 0.54)	**<0.001**
Esthetic medicine procedures, *n* (%)	55 (12.0)	35 (13.5)	20 (10.0)	1.35 (0.81; 2.27)	0.315
Pediatric ophthalmology visits, *n* (%)	258 (56.1)	233 (89.3)	25 (12.6)	7.11 (4.91; 10.28)	**<0.001**

RR—relative risk (Poland vs. UK); CI—confidence interval. Variables compared with Pearson’s chi-squared test. Bold values indicate statistically significant differences (*p* < 0.05).

**Table 3:** Summary of review-related parameters and cross-country comparison.

Variable	Total Group	Poland	UK	MD/RR (95% CI)	*p *
Mean grading (no. of “stars”), median (IQR)	5.00 (4.98; 5.00)	5.00 (5.00; 5.00)	4.97 (4.94; 4.99)	0.03 (0.02; 0.03)	**<0.001 ^1^**
Total number of reviews, median (IQR)	93.00 (50.00; 173.00)	141.00 (77.00; 262.00)	44.00 (25.00; 96.25)	97.00 (65.00; 99.00)	**<0.001 ^1^**
Number of reviews in cohort providing pediatric visits, median (IQR)	1.00 (0.00; 18.00)	14.00 (3.00; 37.00)	0.00 (0.00; 0.00)	14.00 (10.00; 16.00)	**<0.001 ^1^**
Substantive reviews, *n* (%)	425 (92.2)	251 (96.2)	174 (87.0)	1.11 (1.04; 1.17)	**0.001**
Number of substantive reviews, median (IQR)	4.00 (2.00; 8.00)	4.00 (2.00; 10.00)	3.00 (1.00; 6.00)	1.00 (1.00; 2.00)	**<0.001 ^1^**
Mixed reviews, *n* (%)	460 (99.8)	260 (99.6)	200 (100.0)	1.00 (0.99; 1.00)	>0.999 ^2^
Number of mixed reviews, median (IQR)	23.00 (12.00; 47.00)	29.00 (19.00; 56.00)	15.00 (8.25; 33.00)	14.50 (10.00; 17.00)	**<0.001 ^1^**
Soft reviews, *n* (%)	461 (100.0)	261 (100.0)	200 (100.0)	−	−
Number of soft reviews, median (IQR)	57.00 (28.00; 121.00)	**107.00 (54.00; 201.00)**	**26.00 (15.00; 46.75)**	81.50 (58.00; 84.00)	**<0.001^1^**

IQR—interquartile range; MD—mean or median difference (Poland vs. UK); RR—relative risk (Poland vs. UK). Numeric variables compared with Mann–Whitney U test ^1^. Nominal variables compared with Pearson’s chi-squared test or Fisher’s exact test ^2^. Bold values indicate statistically significant differences (*p* < 0.05).

**Table 4:** Factors associated with the number of soft skill-related reviews in univariate and multivariate models.

Variable	Univariate Model				Multivariate Model			
	ß	95% CI for ß	td. ß	*p*	ß	95% CI for ß	td. ß	*p*
Sex, female (vs. male)	51.61	27.92 to 75.30	0.39	**<0.001**	9.08	3.30 to 14.86	0.07	**0.002**
City size, above 100k citizens (vs. capital city)	51.94	22.52 to 81.36	0.40	**0.001**	−	−	−	−
City size, other (vs. capital city)	−1.36	−40.88 to 38.15	−0.01	0.946	−	−	−	−
Professional experience, years	0.71	−0.54 to 1.95	0.05	0.264	−	−	−	−
Scientific title, PhD (vs. MD)	27.16	−1.51 to 55.83	0.21	0.063	−	−	−	−
Scientific title, Professor (vs. MD)	−34.51	−121.36 to 52.35	−0.26	0.435	−	−	−	−
Number of Pubmed publications	-0.58	−1.13 to −0.02	−0.10	**0.041**	−	−	−	−
Public workplace	−76.60	−100.37 to −52.82	−0.58	**<0.001**	−	−	−	−
Surgical procedures	−73.29	−98.18 to −48.41	−0.56	**<0.001**	−6.68	−13.19 to −0.16	−0.05	**0.045**
Esthetic medicine procedures	23.73	−12.68 to 60.13	0.18	0.201	−	−	−	−
Pediatric ophthalmology visits	113.51	91.59 to 135.42	0.86	**<0.001**	−	−	−	−
Mean grading (no. of “stars”)	127.85	27.09 to 228.61	0.12	**0.013**	16.63	−4.41 to 37.66	0.02	0.121
Total number of reviews	0.73	0.71 to 0.74	0.98	**<0.001**	0.71	0.70 to 0.73	0.96	**<0.001**
Country, Poland (vs. UK)	−116.64	−138.48 to −94.80	−0.89	**<0.001**	−8.12	−15.15 to −1.10	−0.06	**0.024**

ß—beta coefficient; CI—confidence interval; std. ß—standardized beta coefficient. Bold values indicate statistically significant differences (*p* < 0.05).

**Table 5:** Characteristics of physicians with high (>100) versus low (≤100) number of patient reviews.

Variable	High Number of Reviews (>100)	Low Number of Reviews (≤100)	MD/RR (95% CI)	*p *
Sex, female, *n* (%)	112 (52.1)	102 (41.5)	1.26 (1.03; 1.53)	**0.029**
City size, *n* (%)				
Capital city	37 (17.2)	64 (26.0)	−	**0.004**
Above 100k citizens	152 (70.7)	137 (55.7)		
Other	26 (12.1)	45 (18.3)		
Professional experience, years, mean ± SD	22.56 ± 9.54	22.82 ± 9.93	0.26 (−2.06; 1.55)	0.782 ^1^
Scientific title, *n* (%)				
Physician	154 (71.6)	192 (78.0)	−	0.194
PhD	57 (26.5)	48 (19.5)		
Professor (full)	4 (1.9)	6 (2.4)		
Number of PubMed publications, median (IQR)	0.00 (0.00; 3.00)	4.00 (1.00; 10.00)	−4.00 (−6.00; −3.00)	**<0.001 ^2^**
Public workplace, *n* (%)	112 (52.3)	171 (69.5)	0.75 (0.65; 0.88)	**<0.001**
Surgical procedures, *n* (%)	128 (59.5)	185 (75.2)	0.79 (0.69; 0.90)	**<0.001**
Esthetic medicine procedures, *n* (%)	24 (11.3)	31 (12.6)	0.89 (0.54; 1.47)	0.768
Pediatric ophthalmology, *n* (%)	169 (78.6)	89 (36.3)	2.16 (1.81; 2.59)	**<0.001**
Number of substantive reviews, median (IQR)	8.00 (3.00; 16.00)	2.00 (1.00; 4.00)	6.00 (4.00; 6.00)	**<0.001 ^2^**
Number of soft reviews, median (IQR)	127.00 (93.50;235.50)	31.00 (16.00;46.00)	96.00 (90.00;111.00)	**<0.001 ^2^**
Country, *n* (%)				
Poland	168 (78.1)	93 (37.8)	−	**<0.001**
UK	47 (21.9)	153 (62.2)		

SD—standard deviation; IQR—interquartile range; MD—mean or median difference (high number of reviews vs. low number of reviews); RR—relative risk (high number of reviews vs. low number of reviews). Numeric variables compared with t-Student ^1^ test or Mann–Whitney U test ^2^. Nominal variables compared with Pearson’s chi-squared test. Bold values indicate statistically significant differences (*p* < 0.05).

**Table 6:** Outcomes of linear regression analysis identifying predictors of high review volume (“top-rated ophthalmologist”: >100 reviews).

Variable	OR	95% CI for OR	*p *	OR	95% CI for OR	*p *
Sex, female (vs. male)	1.54	1.06 to 2.22	0.023	0.53	0.17 to 1.59	0.263
City size, above 100k citizens (vs. capital city)	1.92	1.21 to 3.08	0.006	−	−	−
City size, other (vs. capital city)	1.00	0.53 to 1.87	0.999	−	−	−
Professional experience, years	1.00	0.98 to 1.02	0.781	−	−	−
Scientific title, PhD (vs. physician)	1.48	0.96 to 2.30	0.080	−	−	−
Scientific title, Professor (vs. physician)	0.83	0.21 to 2.96	0.778	−	−	−
Number of PubMed publications	0.98	0.96 to 0.99	0.026	−	−	−
Public workplace	0.48	0.33 to 0.70	<0.001	−	−	−
Surgical procedures	0.49	0.33 to 0.72	<0.001	5.84	1.59 to 25.05	0.011
Esthetic medicine procedures	0.88	0.50 to 1.55	0.661	−	−	−
Pediatric ophthalmology	6.44	4.27 to 9.85	<0.001	−	−	−
Number of substantive reviews	1.26	1.20 to 1.34	<0.001	1.29	1.18 to 1.43	<0.001
Number of soft reviews	1.09	1.07 to 1.11	<0.001	1.14	1.11 to 1.18	<0.001
Country, Poland (vs. UK)	0.17	0.11 to 0.26	<0.001	−	−	−

OR—odds ratio; CI—confidence interval.

## Data Availability

Data are available from the corresponding author upon reasonable request.
